# Probiotics in the Prevention of the Calcium Oxalate Urolithiasis

**DOI:** 10.3390/cells11020284

**Published:** 2022-01-14

**Authors:** Paulina Wigner, Michał Bijak, Joanna Saluk-Bijak

**Affiliations:** 1 Department of General Biochemistry, Faculty of Biology and Environmental Protection, University of Lodz, 90-136 Lodz, Poland; joanna.saluk@biol.uni.lodz.pl; 2 Biohazard Prevention Centre, Faculty of Biology and Environmental Protection, University of Lodz, 90-136 Lodz, Poland; michal.bijak@biol.uni.lodz.pl

**Keywords:** oxalate metabolism, probiotics, kidney stones

## Abstract

Nephrolithiasis ranks third among urological diseases in terms of prevalence, making up about 15% of cases. The continued increase in the incidence of nephrolithiasis is most probably due to changes in eating habits (high protein, sodium, and sugar diets) and lifestyle (reduced physical activity) in all developed countries. Some 80% of all kidney stones cases are oxalate urolithiasis, which is also characterized by the highest risk of recurrence. Frequent relapses of nephrolithiasis contribute to severe complications and high treatment costs. Unfortunately, there is no known effective way to prevent urolithiasis at present. In cases of diet-related urolithiasis, dietary changes may prevent recurrence. However, in some patients, the condition is unrelated to diet; in such cases, there is evidence to support the use of stone-related medications. Interestingly, a growing body of evidence indicates the potential of the microbiome to reduce the risk of developing renal colic. Previous studies have primarily focused on the use of *Oxalobacter* *formigenes* in patients with urolithiasis. Unfortunately, this bacterium is not an ideal probiotic due to its antibiotic sensitivity and low pH. Therefore, subsequent studies sought to find bacteria which are capable of oxalate degradation, focusing on well-known probiotics including *Lactobacillus* and *Bifidobacterium* strains, *Eubacterium lentum*, *Enterococcus faecalis*, and *Escherichia coli*.

## 1. Introduction

Nephrolithiasis is characterized by the presence of insoluble deposits in the urinary tract, so-called stones, that obstruct the proper urine flow. These deposits are a result of increased calcium, oxalic acid, phosphate, urate, and cystine levels in the urine. Unfortunately, the incidence of urolithiasis has dramatically increased over the past 30 years, likely due to environmental changes, including inadequate diet and physical activity [1]. Nowadays, nephrolithiasis ranks third in the list of the most common urological diseases [2]. The incidence of urolithiasis ranges from 7 to 13% in North America, 5 to 9% in Europe, and 1 to 5% in Asia. Nephrolithiasis most often occurs in men aged 40–50 years, and in women aged 50–70 [3,4,5]. Epidemiological data have confirmed that the propensity to form deposits in the urinary tract depends on sex, ethnicity, and geography. Until recently, men were characterized as being 2–3-times more likely to develop kidney stones than women. However, a recent analysis suggested that this disparity is disappearing. Between 1970 and 2000, in Minnesota (the United States), the male to female ratio of kidney stone incidents reduced from 3.1 to 1.3 [6]. This increase in the frequency of nephrolithiasis in women might be due to changes in lifestyle and diet causing obesity, a known risk factor for deposit formation in the urinary tract [7]. In addition to its widespread occurrence, nephrolithiasis is also characterized by a high risk of recurrence. The spontaneous five-year recurrence rate is 35 to 50% following the first renal colic [8]. Moreover, in cases of patients who do not use meta-phylaxis, the relapse rate of secondary deposits is estimated to be 10–23% per year, 50% in 5–10 years, and 75% in 20 years after the first occurrence [9]. Unfortunately, urolithiasis recurrence contributes to the development of several complications, such as pyelonephritis, urinary tract infection, renal insufficiency, and even urinary tract cancer [10,11]. The high prevalence and recurrence of urolithiasis in the general population also contribute to high medical care costs. In the United States, the total cost of healthcare for urolithiasis patients was about $2.1 billion in 2000 [12]. Interestingly, it has been estimated that by 2030, this figure will increase by $1.24 billion per year because of the rising prevalence of obesity and diabetes [13].

As noted above, deposits in the urinary tract can vary in their chemical composition; however, 80% of stones are calcium-based, 9% uric acid-based, 10% struvite-based, and 1% are cystine stones [14]. Among all types of urinary calcium stones, 50% are calcium oxalate deposits, 5% are calcium phosphate, and 45% are a mixture of both kinds [15]. Kidney stone formation is a complex and multifactorial process that depends on intrinsic (age, sex, and heredity) and extrinsic (geography, climate, dietary, mineral composition, and water intake) factors [16]. Common risk factors for the development of nephrolithiasis are presented in Table 1.

Importantly, disturbing epidemiological data suggest the existence of additional risk factors for the development of urolithiasis. In the last 15 years, the incidence of kidney stones in the United States has doubled, making its prevalence equivalent to that of diabetes. This increase was mainly recorded among children and women. These alarming changes in the incidence of urolithiasis have contributed to the search for new determinants of nephrolithiasis. Additionally, antibiotics and their impact on the composition of the intestinal and urinary microbiome have been examined. Epidemiological analyses have shown that 30% of patients are prescribed at least one antibiotic per year, and previous studies have linked oral antibiotic use with increased prevalence of kidney stones among children and adults [22,23]. Interestingly, Ferraro et al. [24] confirmed the relationship between exposure to antibiotics and changes in the chemical composition of urine. Exposure to antibiotics for at least two months resulted in a decrease in urine pH and urinary citrate levels, suggesting that there is a complex correlation between the gut and urinary tract in kidney stones [24]. These results were confirmed by Stern and colleagues [25]. Genetic hypercalciuric stone-forming rats were characterized by decreased urine pH and urinary citrate levels [25]. Miller et al. [26] confirmed previous reports which found that the multispecies bacterial network, containing, e.g., *Ruminococcus*, *Oscillospira*, *Desulvovibrio* and *Methanobrevibacter*, maintains oxalate homeostasis [26]. Thus, the present study aimed to identify unique changes in the gut microbiome of patients with kidney stones compared to controls. The obtained results showed that *Bacteroides* was 3.4 times more abundant in the kidney stone group compared to the control, while *Prevotella* was 2.8 times more abundant in the control group compared to the kidney stones group [27]. Thus, the presented results suggest the existence of an entero-renal axis as the pathway between the gut microbiome, gut metabolites, and urine chemistry, which determines human health and disease [24].

**Figure 1:**
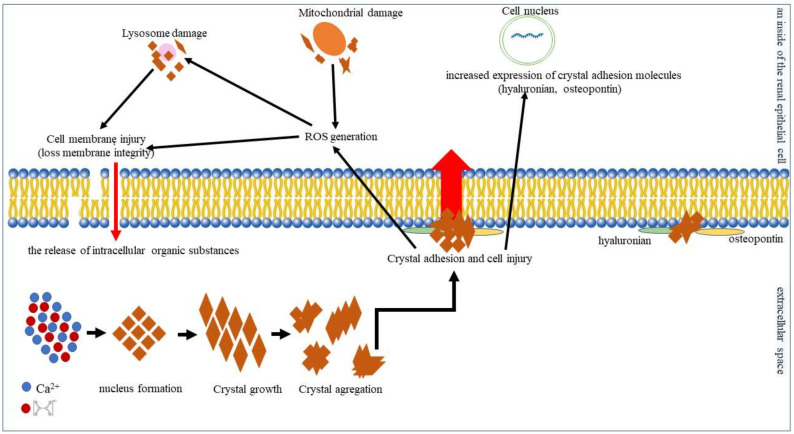
In supersaturated urine, calcium and oxalate combine to form initially microscopic insoluble crystals. This is the first stage of deposit formation (nucleus formation). Then, individual microcrystals combine into larger forms (crystal growth), which, in turn, can aggregate together to form large, stable deposits (crystal aggregation) [34,35]. In the next stage, the crystals interact with the cells of the renal tubular epithelium. Crystal–cell interaction causes the movement of crystals from the basolateral side of cells to the basal membrane [36]. Injured cells release substances such as the kidney fragment of prothrombin−1 or other anionic proteins that induce crystal agglomeration [37]. Moreover, injured cells can invert their cell membrane, which is anionic to the urinary environment and acts as a place of crystal adhesion. The inverted cell membrane makes it easy for other crystals to attach [38]. Exposure to calcium oxalate crystals induces oxidative stress in renal epithelial cells. In addition, calcium oxalate influences the composition and function of the renal epithelial cell membrane. Calcium oxalate crystals destroy tight junctions and the polarity of the cell membrane that carries the components of the basolateral or tight junction region to the apical surface of the cell, which, in turn, leads to rupture of the cell membrane and the release of intracellular organic substances. Damaged tubular epithelial cells also show increased expression of crystal adhesion molecules such as hyaluronan, osteopontin, and CD44, which promote crystal adhesion and retention. Endocytosed crystals adversely affect mitochondrial function, causing abnormality in the respiratory chain and increasing the mitochondrial production of reactive oxygen species (ROS), which may damage, and induce apoptosis of, renal epithelial cells [39].

Kidney stones form when urine contains more dissolved material (such as calcium, oxalate, and uric acid) than can be dissolved under normal circumstances [28]. The mechanism of deposit in the urinary tract is presented in Figure 1. The level of calcium in the urine depends on the calcium content in the diet, as well as on the correct function of the metabolic pathways; e.g., with low calcium intake, increased calcium level in the urine may be a consequence of increased bone resorption. In turn, urinary oxalate comes mostly from endogenous metabolic processes, although an additional source may be the increased absorption of oxalate in the intestine with concomitant higher calcium intake [29,30]. Interestingly, dietary oxalate may account for almost 50% of the daily oxalate excreted in urine. Moreover, calcium oxalate stones have an increased propensity to form in the presence of elevated oxalate, even without elevated calcium [31]. Thus, most research efforts to reduce the recurrence of kidney stones have focused on reducing the amount of oxalate consumed. Currently, beyond changes to diet, there is no effective way to lower urinary oxalate level in stone formers. An analysis of fecal samples showed that patients with recurrent idiopathic calcium stones exhibited lower fecal microbial diversity than controls. In particular, these samples were characterized by low levels of bacteria capable of oxalate degradation [32]. Therefore, previous studies have suggested that probiotic bacteria, especially *O. formigenes*, *Lactobacillus* spp. and *Bifidobacterium* spp., which can degrade dietary oxalate in the gastrointestinal tract allowing it to be absorbed, may help reduce urinary oxalate levels [33].

## 2. *Oxalobacter formigenes* 

### 2.1. Characteristics of Oxalobacter formigenes

*Oxalobacter formigenes* comprises immobile, nonspore-forming, obligate, anaerobic, Gram-negative rods which inhabit the human colon and ruminant rumen. It was discovered by Allison and coworkers in 1985 [40]. The characteristics of *O.* *formigenes* are presented in Table 2. Due to oxalate use as the sole source of carbon and energy, *O. formigenes* is called a specialist oxalotroph. Thus, this bacteria can degrade ingested oxalate and reduce intestinal absorption, as well as stimulating the excretion of oxalate from the colon, thereby providing protection from hyperoxaluria, when it presents in the gut microflora [41,42]. It has been assumed that the number of *O. formigenes* cells per gram of stool is 10^7^, and the efficiency of the oxalate degradation process in this environment has been estimated to be 0.1–4.4 nmol per hour per g of stool [43]. However, previous studies have shown that the prevalence of *O. formigenes* varies among the general population. One epidemiological study suggested that *O. formigenes* colonization is associated with the age of the individual, reaching 100% detection in children, and only 40–60% in adults [44]. Interestingly, Sidhu et al. [45] found that feces collected from newborns and infants less than nine months old showed an absence of *O. formigenes*, but that this bacterium was detectable in the feces of children of crawling age. Almost all children between six and eight years old were colonized, but the colonization rate reduced in older children to around that found in the adult population (around 70%) [45]. This variation in *O. formigenes* colonization may be due to high bacterial sensitivity to antibiotics, the use of which is more common among the older population. Previous studies showed that a significantly reduced level of *O. formigenes* gut colonization was associated with increased use of antibiotics and other drugs, particularly in the treatment of cystic fibrosis, inflammatory bowel disease, Crohn′s disease, and jejunoileal bypass surgery [44,45].

**Figure 2:**
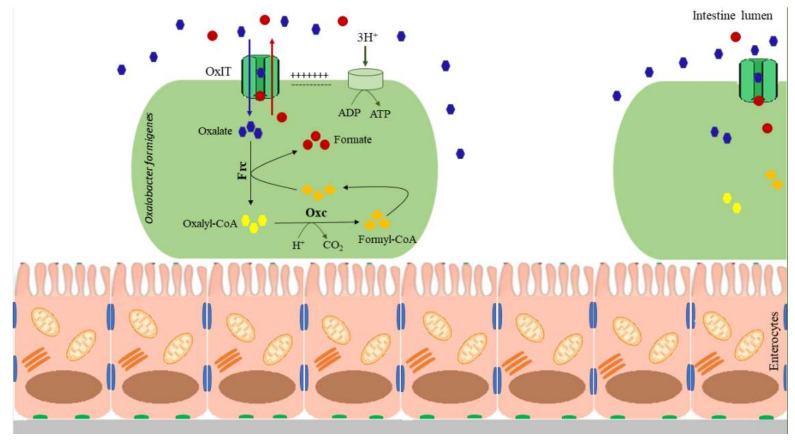
Oxalate metabolism by *Oxalobacter formigenes.* OxIT—oxalate transporter, Oxc—oxalyl-CoA decarboxylase, Frc—formyl-CoA transferase.

The ability of *O. formigenes* to metabolize oxalate may be attributed to the presence of two enzymes, i.e., formyl-CoA-transferase and oxalyl-CoA-decarboxylase. *O. formigenes* takes up oxalate via the OxIT transporter encoded by the *OxIT*. This transport relies on the extracellular uptake of oxaloacetate in return for the formate released from the cell. In bacterial cells, formyl-CoA transferase (encoded by *fcr*) activates the oxalate by adding coenzyme A molecules with the formation of oxalyl-CoA. Then, oxalyl-CoA is decarboxylated by oxalyl-CoA decarboxylase (encoded by *oxc*). The oxalyl-CoA decarboxylase produces CO_2_ and formate, which are used by OxIT to transport oxalate. Oxalate decarboxylation generates a proton pump gradient that induces ATP production, coupling with oxalate–formate transport [46]. Oxalate metabolism is presented in Figure 2.

### 2.2. Oxalobacter formigenes and Kidney Stone Course

Previous analyses have shown that up to 40% of the oxalate present in the urine may come from the intestines. These results suggest that limiting the absorption of oxalate in the gut may be important for minimizing the risk of developing or recurring urolithiasis [47]. *O. formigenes* regulates intraluminal oxalate availability by degrading dietary oxalate, using oxalate as an obligatory energy source, thereby reducing intestinal oxalate absorption and urinary excretion [48]. Consequently, oxalate degradable *O. formigenes* limits the availability of oxalate for intestinal absorption. However, the intestinal colonization by *O. formigenes* does not affect other factors related to the development of urolithiasis, such as concentration of calcium, uric acid, sodium, citrate and magnesium in the urine [47]. Hatch et al. [48] suggested that *O. formigenes* may also interact physiologically with the colonic mucous membrane by inducing enteric oxalate secretion, leading to additional reduction of urinary oxalate excretion [48]. This ability to break down oxalate and reduce urinary oxalate excretion suggests the possible clinical application of *O. formigenes* in the treatment and prevention of oxalate nephrolithiasis. Unfortunately, a composition analysis did not confirm the presence of *O. formigenes* in probiotics available on the market which state this bacteria among their ingredients. This suggests that the production of products containing *O. formigenes* is challenging [49]. The difficulty in making probiotics containing *O. formigenes* may be due to its sensitivity to oxygen and low pH, strict oxalate demand for growth, and intolerance to the processes which are commonly involved in the manufacture, storage, and distribution of probiotic products. However, recent results suggested that individual strains of *O. formigenes* may differ in their resistance to the above-mentioned stress stimuli [50]. A molecular analysis showed that some *O. formigenes* strains can tolerate a lack of calcium oxalate. Prolonged starvation for some bacteria results in growth advantage in the stationary phase (GASP). The GASP phenotype results from stable mutations that provide an improved ability to survive during starvation and temporarily obtain energy from the transformation of other compounds. Similarly, some *O. formigenes* strains have shown the ability to temporarily acclimatize to aerobic conditions due to the presence of superoxide dismutase [50]. Therefore, *O. formigenes* is an extensively studied bacterium for the prevention of urolithiasis, and extensive research has been carried out in search of strains which are resistant to the production conditions of probiotics. On the other hand, a previous study suggested that the way *O. formigenes* is administered determines its effectiveness in terms of colonizing the gut. Successful and long-lasting colonization has been observed in healthy adults where *O. formigenes* was formulated as a spread on a turkey sandwich with a sodium oxalate load [51], while colonization of the intestines of *O. formigenes* provided either in lyophilized form or as a frozen cell paste to patients with primary hyperoxaluria was unsuccessful [52]. Thus, further detailed studies on the sensitivity of individual *O. formigenes* strains to production conditions are necessary.

Previous studies showed that rats fed a diet containing 0.5–1.5% oxalate which became colonized with *O. formigenes* were characterized by decreased urinary oxalate levels, compared with noncolonized rats on the same diet [48,53,54]. It should be remembered that the dose of *O. formigenes* administered orally limits the speed and effectiveness of the final gut colonization, due to the low pH of the stomach, which is considered to be the main barrier to the survival of ingested bacteria in the gut. Therefore, a comparative analysis of different doses of *O. formingens* (1 × 10^3^, 1 × 10^5^, 1 × 10^7^, 1 × 10^9^ CFU), given to subjects on an oxalate-rich diet, showed that the level of the urinary oxalate extraction decrease was directly proportional to the dose of bacteria [54]. Similarly, human studies confirmed that daily intake of *O. formigenes* can lead to reduced urinary oxalate levels in patients on a diet which is high in calcium oxalate [51,52]. The reduced urinary oxalate levels in subjects on an oxalate-rich diet confirmed in vivo that *O. formigenes* may be an effective probiotic in preventing the formation of deposits in the urinary tract. Unfortunately, despite achieving statistically significant differences in both of the presented studies, the results were characterized by a low degree of certainty, since the study groups consisted of a small number of patients—four in the case of Duncan’s study and nine in Hoppe’s study [51,52]. On the other hand, a study involving 35 first-time calcium oxalate stone formers and ten control subjects confirmed that urolithiasis patients were characterized by lower levels of *O. formigenes* gut colonization than healthy volunteers. Intestinal *O. formingens* was detected in only 26% of stone formers compared with 60% of the control group. Although the obtained results showed that the average urinary oxalate excretion by both groups was similar (38.6 mg/day vs. 40.8 mg/day), among patients with stones, *O. formigenes*-negative patients were characterized by higher urinary oxalate concentrations than those testing positive, which clearly confirms the role of *O. formigenes* in oxalate degradation in vivo [49]. Similarly, Siener et al. [55] also confirmed that patients with urolithiasis are characterized by a low level of *O. formigenes* colonization. Among 37 patients, only 11 were *O. formigenes*-positive. Additionally, the mean plasma oxalate concentration was more than threefold higher in patients without *O. formigenes* than patients with this bacteria [55]. Kaufman et al. [56] also showed that adults with kidney stone disease were characterized by less intestinal tract *O. formigenes* colonization than healthy subjects, and that urinary oxalate exertion might depend upon bacterial colonization [56]. This was the first large study (247 patients with recurrent calcium oxalate stones and 259 controls) conducted under controlled conditions to reliably confirm the potential of *O. formigenes* in the prevention of urolithiasis. Interestingly, additional analyses by Kaufman and colleagues showed that the prevalence of *O. formigenes* colonization in the studied population depended on the rate of recurrent stone incidence [56]. Siener and colleagues [55] found that *O. formigenes* colonization was 67, 33, and 16% in patients with one, two to four, and more than four stone episodes, respectively [55]. Thus, gut colonization of *O. formigenes* might lead to a 70% reduction in the risk of recurrent stone disease [55,56], and intestinal recolonization may be effective in reducing the development and recurrence of urolithiasis.

Unfortunately, an animal study reported that *O. formigenes* colonization is transient. Five to 10 days after the oxalate withdrew, the fecal population of *O. formigenes* was undetectable by polymerase chain reaction (PCR). However, as mentioned above, the colonization efficiency may also be influenced by the selection of an appropriate strain of *O. formigenes*, i.e., one which is resistant to the presence of oxygen and low pH, as well as the selection of an appropriate method of administration. On the other hand, animals with chronic hyperoxaluria on an oxalate-rich diet showed decreased urinary oxalate within two days of initiating *O. formigenes* supplementation [54]. These results demonstrate the high effectiveness of *O. formigenes* in reducing urinary oxalate levels. However, the temporary colonization of the intestines with this bacteria means that in order to achieve a lasting reduction in the risk of developing urolithiasis, continuous or repeated treatment may be necessary. Moreover, epidemiological studies showed that *O. formigenes* prevalence in developed countries is lower than in developing countries. *O. formigenes* occurrence in the United States population was estimated at 31–38%, while in India, this figure was 60–77% level [57,58,59]. These differences in the levels of *O. formigenes* gut colonization among individuals may be due to different medical practices. Studies conducted so far have shown a high sensitivity of *O. formigenes* to antibiotics use [53,60]. A comparative analysis showed that *O. formigenes* prevalence in the Amerindians of the Yanomami-Sanema and Yekwana ethnic groups in Venezuela and the Hadza in Tanzania was higher than in adults from the USA. The analyzed tribes were characterized by a general lack of contact with modern medical practices, including antibiotic therapy [61]. A human study confirmed that *Helicobacter pylori* eradication using antibiotics caused loss of *O. formigenes* colonization for at least six months post-treatment [60,62]. Therefore, antibiotics may affect the number of bacteria in the gut, which, in turn, translates into an increased risk of developing kidney stones in people frequently receiving antibiotics. In addition to antibiotics, the level of *O. formigenes* colonization also depends on diet. Jiang et al. [63] found that the amount of calcium and oxalate in the diet affects the number of bacteria. Eleven *O. formigenes*-positive and 11 *O. formigenes*-negative patients were administered diets with controlled calcium and oxalate contents. In the first phase, dietary oxalate intake was varied (50 mg daily for the first week, 250 mg for the second week and 750 mg for the third week), while calcium consumption was fixed at 1000 mg daily. For the second phase, dietary calcium intake was varied (400 mg daily for the first week, 1000 mg for the second week and 2000 mg for the third week), while oxalate was fixed at 250 mg daily. Tests showed that the mean *O. formigenes* level increased 10-fold when dietary oxalate increased 15-fold, while there was a decrease in *O. formigenes* content with increasing calcium intake [63]. The sensitivity of *O. formigenes* to selected antibiotics and diet is a significant factor limiting the use of this bacterium in the prevention of urolithiasis. Nevertheless, the development of an effective dosing regimen of *O. formigenes* after antibiotic therapy and the modification of dietary habits may positively affect the effectiveness of *O. formigenes* in preventing the formation of deposits in the urinary tract [60,61,63].

However, studies on the differentiation of the composition of the intestinal microflora in patients with urinary stones have shown that antibiotic therapy promotes the development of urolithiasis not only due to the reduction of *O. formigenes* colonization, but also by modifying the composition of the entire intestinal microflora. The gut microbiome is a complex ecosystem, harboring a large number of species, including bacteria metabolizing oxalate that build complex functional networks among each other. Typically, the gut plays an important role in the metabolism of oxalate, which lowers the risk of kidney stone formation. The relationship between the gut and kidneys is called the gut–kidney axis [64]. The importance of the gut–kidney axis in the development of urolithiasis is emphasized by the fact that hyperoxaluria, observed in the course of urolithiasis, may increase intestinal permeability. Disturbances in the intestinal microflora that accompany urolithiasis can induce damage to the intestinal barrier. Increased intestinal permeability promotes the translocation of bacterial waste products that induce a response to the developing inflammation in the intestinal and urinary tract [65]. A previous analysis showed that people who form kidney stones were characterized by microbial dysbiosis, including an abnormal abundance of eubacteria, archaea, eukaryotes and even fungi. In addition to *O. formigenes*, research to date has also indicated the insufficiency of *Ruminococcus* and *Oscillospira* as causes of abnormal oxalate homeostasis in the gut [66]. A metagenomics analysis showed that the fecal microbiomes of stone formers exhibited reduced biodiversity and numbers. Patients with kidney stones were characterized by selective depletion of *Faecalibacterium prausnitzii*, *Prevotella*, *Dialister*, *Dorea*, and *Enterobacter*. Moreover, high *Sutterella*, *Veillonella*, and *Peptococcus* abundance were significantly correlated with urinary oxalate excretion [64]. Another study showed that changes in the microbiome which may contribute to the onset of kidney stones were associated with a depletion of *Lacttobacillus* and an increased amount of pathogenic *Enterobacteriaceae* [66].

Therefore, even effective colonization of the intestines by *O. formigenes* in patients with impaired intestinal microbiomes may be ineffective in the prevention of urolithiasis, and the proper functioning of the gut–kidney axis may not depend only on the presence of *O. formigenes*. Importantly, the discrepancies presented above regarding the effectiveness of the use of preparations containing *O. formigenes* in the prophylaxis of urolithiasis may result from disturbances of the entire intestinal microflora, and not just intestinal colonization by *O. formigenes*, although this requires further analysis [64].

Interestingly, due to the excellent ability of *O. formigenes* to break down oxalate, this bacterium in the form of an oral probiotic has been the subject of clinical trials. Phase I/II clinical trials demonstrated that the twice-daily treatment for eight weeks with the Oxabact^®^ OC5 formulation containing *O. formigenes* did not reduce urinary oxalate excretion in patients diagnosed with primary hyperoxaluria. However, additional analysis of the ratio of urinary oxalate excretion to urinary creatinine excretion showed a significant increase after eight weeks of treatment in the treated group compared with the placebo group. Unfortunately, even though phase II/III clinical trials showed that the 24-week treatment with Oxabact™ OC3 was safe and well-tolerated in patients with primary hyperoxaluria, no effects were noted of the treatment on urinary oxalate levels or on the ratio of urinary oxalate excretion to urinary creatinine excretion [67,68,69].

A summary of previous studies using *O. formigenes* for the prevention of urolithiasis is presented in Table 3.

## 3. *Lactobacillus* spp.

### 3.1. Characteristics of Lactobacillus spp.

*Lactobacillus* species are Gram-positive, nonspore-forming rods occurring in high numbers in the human gut. The characteristics of *Lactobacillus* spp. are presented in Table 4. *Lactobacillus* spp. is generally considered to be an oxygen-tolerant anaerobic with fermentative metabolism. *Lactobacillus* are homofermentative or heterofermentative bacteria. *Lactobacillus*, as a homofermentative bacterium, metabolizes hexoses through the glycolysis pathway to lactate, while as a heterofermentative bacterium, it metabolizes hexoses through the phosphoketolase pathway to lactate, CO_2_, and acetate or ethanol [70]. Moreover, *Lactobacillus* species are called “generalist oxalotrophs” since, in contrast to *O. formigenes*, they can grow using a range of energy sources in addition to oxalate [71]. These bacteria are approved as generally regarded as safe (GRAS) for human consumption by the United States Food and Drug Administration (FDA). Thus, they have been used widely as probiotics to promote human health [72]. *Lactobacillus* spp. is a crucial component of the gut microbiome which is involved in the prevention and treatment of irritable bowel syndrome, inflammatory bowel disease (*L. plantarum*), acute diarrhea (*L. casei*), urogenital disease, dental caries, and food hypersensitivity (*L. reuteri*), as well as in the prevention of infections such as *H. pylori* and *Clostridium difficile* (*L. casei*, *L. reuteri*) [73,74,75,76,77]. 

*L. casei* improves the body’s response to glucose and weight gain, and thus, may be used in type II diabetes therapy [78]. Interestingly, a growing body of evidence suggests that certain probiotic species, including *L. casei*, *L. helveticus*, and *L. rhamnosus*, can modulate mood, increase stress resilience, and alleviate anxiety [79]. Recent studies also indicate the participation of *Lactobacillus* spp. in the prevention of kidney stones [80].

### 3.2. Lactobacillus and Kidney Stone Prevention

Previous in vitro studies showed that only some of *Lactobacillus* and *Bifidobacterium* strains have *oxc*-like genes which provide oxalate degradability. Therefore, the various strains of these species showed highly variable oxalate degrading capacity [81,82,83]. A molecular analysis showed that *frc* and *oxc* genes encoding functional oxalate-degrading enzymes were identified in *L. acidophilus* NCFM and *L. gasseri* AM63T. *Oxc* and *frc* expressions were induced as an operon in the presence of oxalate under acid conditions [71,84]. Additionally, Murphy et al. [85] showed that among the various analyzed strains, only *L. animalis* 223C, *L. animalis* 5323, *L. murinus* 1222, and *L. murinus* 3133 showed the ability to decompose oxalate in vitro. However, an animal study confirmed that among the studied strains, only *L. animalis* 223C and *L. animalis* 5323 reduced oxalate excretion in vivo [85]. These results prove that *Lactobacillus* strains show the ability to degrade oxalate, and thus, their potential for clinical application. Moreover, the mismatch between in vitro and in vivo test results emphasizes that in vitro oxalate degradation does not reflect the ability of bacteria to metabolize oxalate in vivo after intestine colonization, and that proving the efficacy of a given bacterium in a clinical setting requires long-term research. A subsequent in vitro study confirmed that *L. acidophilus*, *L. gasseri*, *L. salivarius* AB11, *L. fermentum* TY12 and five strains of *L. fermentum* sp., *Weissella confuse,* and *W. cibaria* showed oxalate degrading ability. However, only *L. acidophilus*, *L. gasseri,* and *L. salivarius* AB11 were characterized by good adhesion to epithelial cells and strong antimicrobial activity. Moreover, these *Lactobacillus* strains also exhibited resistance to kanamycin, rifampicin, and ampicillin, but were sensitive to chloramphenicol and erythromycin. Thus, these strains may be good probiotic candidates for preventing hyperoxaluria, also in patients who have recently been treated with antibiotics [86]. Additionally, Giardina et al. [87] found that *L. plantarum* PBS067 and *L. acidophilus* LA-14 exhibited not only the ability to break down oxalate, but also may modulate the release of inflammation mediators associated with oxalate accumulation in the human gastrointestinal tract. 

*L. plantarum* PBS067, *B. breve* PBS077 and *B. longum* PBS108 increased the production of IL-4, IL-10, IFN-γ and IL-12p70. Therapy with these probiotic bacteria not only contributed to the active degradation of oxalate, but also reduced the number of inflammatory events associated to with oxalate accumulation by stimulating cytokine production. Therefore, this therapy may be recognized as an innovative biological tool for the prevention and therapeutic treatment of kidney stones [87].

Screening different probiotic strains for their in vitro ability showed that *L. paracasei* LPC09 (DSM 24243) was characterized by the highest level of oxalate degrading activity (breakdown 68.5% of 10 mM ammonium oxalate). *L. gasserii* LGS01 (68.4%), *L. gasseri* LGS02 (66.2%), *L. acidophilus* LA07 (54.3%), and *L. acidophilus* LA02 (51.4%) showed a slightly lower ability to decompose ammonium oxalate in vitro. Among other studied bacterial strains, *L. reuteri* LRE03, *L. reuteri* LRE02, and *L. rhamnosus* LR06 degraded over 20% of the oxalate present in the medium. An indispensable advantage of these probiotic bacteria is that they have been classified as GRAS for human consumption by FDA. Therefore, it is only necessary to confirm their ability to degrade oxalate in vivo by clinical tests. This could allow the use of the tested probiotics to be extended to the prophylaxis of urolithiasis [83]. A subsequent evaluation of oxalate degradation in vitro confirmed that *L. acidophilus*, *L. plantarum*, *L. brevis*, *Streptococcus thermophilus*, and *Bifidobacterum infantis* may be used as potential probiotic strains. *L. acidophilus* was characterized by the highest percentage breakdown of 10 mM ammonium oxalate (11.8%) and *L. brevis* the lowest (0.9%). *S. thermophilus* and *B. infantis* degraded 2.3% and 5.3% ammonium oxalate, respectively [80]. Other bacteria which are capable of reducing intestinal oxalate absorption are *L. casei* HY2743 and *L. casei* HY7201 [88]. An animal study confirmed that rats treated with *L. casei* HY2743 and *L. casei* HY7201 showed decreased urine oxalate excretion compared to the group without *Lactobacillus* supplementation. Moreover, a microscope examination of rat kidneys confirmed a lower abundance of crystals in the group with *L. casei* HY7201 therapy than controls, confirming the effectiveness of the tested probiotics in preventing the formation of stones in the urinary tract [89].

Other in vivo studies showed that the administration of probiotic *Lactiplantibacillus plantarum N-1* (LPN1) for four weeks prior to the initiation of hyperoxaluria, induced by ethylene glycol, inhibited the development of oxalate deposits in the kidney. Additionally, rats subjected to preventive intervention with LPN1 showed reduced expression of oxalic acid and renal osteopontin and CD44 in the urine, as well as improved ethylene glycol-induced enteritis and barrier function due to reduced LPS (lipopolysaccharide) and TLR4 (Toll-like receptor 4)/NF-κB (nuclear factor kappa-light-chain-enhancer of activated B cells) signaling in serum and upregulation of claudin -2 of the colon. Additionally, they showed increased production of short-chain fatty acids (SCFA) and an abundance of beneficial SCFA-producing bacteria, mainly from the *Lachnospiraceae* and *Ruminococcaceae* families. As such, it was observed that the LPN1 probiotic can prevent ethylene glycol-induced hyperoxaluria by regulating the gut microflora and improving gut barrier function [90].

Similar to *O. formigenes, Lactobacillus* strains have been tested extensively in clinical trials. Due to the fact that studies conducted so far have shown that multistrain probiotics seem to be more effective than single strains, clinical studies on probiotics with a prophylactic potential against urolithiasis have mainly included mixtures of various probiotic bacteria. However, it should be remembered that it is currently unclear whether the improved efficacy of the bacterial blend is due to synergistic interactions between strains or a consequence of the higher probiotic dose used in some studies [91]. Campieri et al. [80] found that a mixture of freeze-dried *Lactobacillus* strains (*L. acidophilus*, *L. plantarum*, and *L. brevis*), *B. infantis*, and *S. thermophilus*, administered as a daily dose at 8 × 10^11^ CFU, caused a significant reduction in the urinary excretion of oxalate, i.e., by about 40% (55.5 ± 19.6 mg/24 h reduced to 35.5 ± 15.9 mg/24 h), in six patients with idiopathic calcium oxalate urolithiasis and mild hyperoxaluria after four weeks probiotic therapy [80]. Similarly, in the case of patients with chronic fat malabsorption, calcium oxalate stones, and hyperoxaluria, therapy using an Oxadrop^®^ probiotic preparation (VSL Pharmaceuticals, Rome, Italy) containing *L. acidophilus*, *B. infantis*, *S. thermophilus*, and *L. brevis* (mixed in a 1:1:4:4 weight and prepared as a granulate contained 2 × 10^11^ bacteria/1 g preparation) was associated with decreased urinary oxalate excretion. Additionally, urinary oxalate excretion was dependent on the dose of the probiotic taken; ten patients received increasing doses of Oxadrop^®^—4, 8, and 12 g—for one month each (the total duration of therapy was three months). Taking 4 g of Oxadrop^®^ per day reduced urinary oxalate excretion by 19%; this increased to 24% when 8 g per day were administered [92]. Thus, it would seem that Oxadrop^®^ can effectively reduce the risk of further attacks of renal colic. On the other hand, in a randomized, placebo-controlled trial, no effect of Oxadrop^®^ on urinary oxalate excretion was observed in mildly hyperoxaluric stone formers after 28 or 56 days of probiotic therapy [93,94]. Additionally, Lieske et al. [93] found that Oxadrop^®^ and Agri-King Synbiotic (Fulton, IL, USA) containing *Enterococcus faecium*, *Saccharomyces cerevisiae* subsp. *Boulardi*, and *S. cerevisiae* did not influence urinary oxalate levels in patients on a restricted oxalate diet. The obtained results suggested that diet was more effective at preventing the formation of urinary stones than the tested probiotics [93]. Similarly, in the case of a five-week therapy with the Oxadrop^®^ preparation, a daily dose of 8 × 10^11^ CFU (4g/day) showed no effect on urinary and plasma oxalate levels in 20 healthy volunteers who were on an oxalate-rich diet for six weeks [95]. These discrepancies may result from the strictness of the test qualification procedure and the method of controlling the course of the tests. Ferraz et al. [96] showed that another lactic acid bacteria mixture containing *L. casei* and *B. breve* might reduce urinary oxalate excretion in seven stone-forming patients; however, the extraction level may be dependent on dietary oxalate intake, i.e., with low oxalate intake, the reduction in urinary oxalate levels may not be as significant as in patients on an oxalate-rich diet [96]. The beneficial effect of *Bifidobacterium* spp. and *Lactobacterium* spp. was also confirmed in studies on VSL#3^®^ (Sigma-Tau pharmaceuticals, Inc., Gaithersburg, MD, USA). This probiotic supplement consists of freeze-dried live lactic acid bacterial culture, including *Streptococcus thermophilus*, three strains of *Bifidobacterium* species (*B. breve*, *B. longum*, and *B. infantis*), and four strains of *Lactobacillus* species (*L. acidophilus*, *L. plantarum*, *L. paracasei*, and *L. delbrueckii* subsp. *Bulgaricus*). Each sachet of VSL#3^®^ contains 450 billion live bacteria. The daily ingestion for four weeks of one packet of VSL#3^®^ by patients with high oxalate absorption levels led to the reduction of gastrointestinal oxalate absorption, and thus, could decrease the risk of kidney stones [97]. Furthermore, Al-Wahsh et al. [97] confirmed that eleven healthy nonstone formers on a high oxalate diet (176 mg oxalate per day) after VSL#3^®^ therapy were characterized by reduced urinary oxalate and increased oxalate absorption. However, a comparative analysis of the effectiveness of two different doses of the preparation (one and two sachets) showed no significant difference [97]. It should be noted that studies on the beneficial effects of VSL#3^®^ were limited to a small research group. Therefore, it is necessary to conduct a large randomized and controlled trial to confirm the results obtained so far.

A summary of previous studies on the use of *Lactobacillus* spp. in urolithiasis prevention is presented in Table 5.

## 4. *Bifidobacterium* spp.

### 4.1. Characteristic of Bifidobacterium

*Bifidobacterium* was first isolated in 1899 from the feces of breast-fed infants [98]. *Bifidobacterium* species are Gram-positive, nonspore-forming coryneform rods occurring in high numbers in the human gut. The characteristics of *Bifidobacterium* spp. are presented in Table 6. These bacteria are approved as GRAS for human consumption by the FDA. Thus, they are used widely as probiotics to promote human health [99]. Numerous studies have found that the presence of *Bifidobacterium* in the human large intestine provides many human health benefits. *Bifidobacterium* may be useful for constipation treatment in elderly individuals who suffer from long colonic transit times [100]. The addition of large numbers of lactose-digesting cultures, including *Bifidobacterium* spp., to dairy products, may also contribute to alleviating the symptoms of lactose intolerance [101]. Interestingly, some *Bifidobacterium* spp. shows the ability to magazine cholesterol in their cell membranes. Therefore, these bacteria, including *B. animalis* subsp. *Lactis*, and *L. acidophilus*, may contribute to the reduction of plasma cholesterol [102]. Additionally, previous studies showed that *Bifidobacterium* may be involved in oxalate degradation in the gastrointestinal tract. Oxalate degradation by some strains of *Bifidobacterium* is possible due to oxalyl-coenzyme A (CoA) decarboxylase activity encoded by *oxc*, which is key to the oxalate degradation process, and catalyzes the decarboxylation of oxalyl-CoA to formyl-CoA [103].

### 4.2. Bifidobacterium and Kidney Stone Prevention

In addition to well-known *Lactobacillus* probiotics, other probiotic strains of *Bifidobacterium* have shown the ability to degrade oxalate. A molecular analysis identified *oxc* and *frc* homologues in a few *Bifidobacterium* species, including *B. animalis* subsp. *Lactis*, *B. dentium*, *B. gallicum*, *B. pseudocatenulatum*, and *B. pseudolongum*, suggesting that these bacteria may have the ability to break down calcium oxalate. In vitro studies showed that among 12 studied bacterial strains, *B. lactis* DSM 10140, isolated from yoghurt, showed the highest oxalate-degrading activity (degrading 60.6% of available oxalate). Other bacterial strains with high oxalate degradation activity were *B. adolescentis* MB 238 (57% of available oxalate), *B. animalis* ATCC 27536 (49%), *B. breve* MB 283 (37.8%), *B. longum* MB 282 (35.2%), and *B. longum* ATCC 15707 (35%). The strain analysis also showed that the oxalate-degrading ability of several *Bifidobacterium* species was strain-specific. *B. breve* MB 283 degraded 37.8% of available oxalate, whereas *B. breve* MB 151 degraded only 1% [103]. Interestingly, in the case of *B. breve* PBS077 and *B. longum* PBS078, Giardina et al. [87] reported that these bacteria could degrade oxalate as well as modulate the immune response associated with oxalate accumulation in the gastrointestinal tract by modulating the production of pro- and anti-inflammatory cytokines. This immunomodulation was found to contribute to a reduction of inflammatory events associated with oxalate accumulation [87]. In another study, screening different *Bifidobacterium* strains for their in vitro ability to metabolize oxalates showed that *B. breve* BR03 degraded 28.2% of oxalate, *B. animalis*—27.7%, *B. longum* BL03—25.3%, and *B. lactis* BA05—15.5% [83]. Another *Bifidobacterium* species capable of degrading oxalate in vitro is *B. infantis*, which showed about 5% degradation of available oxalate. Moreover, unlike other probiotic bacteria tested (*L. acidophilus*, *L. plantarum*, *L. brevis*, and *S. thermophilus*), only *B. infantis* showed good degrading activity as well as rapid growth in an oxalate-containing medium [80]. Thus, *B. infantis* can more easily colonize the intestines of patients who are prone to the formation of urinary stones. Moreover, the use of a probiotic mix which has a high oxalate degradation activity but slow growth under conditions of high oxalate concentration may contribute to the rapid enhancement of oxalate degradation [80]. Therefore, when looking for bacteria among known probiotics which are capable of decomposing oxalate, it should be remembered that the selected strains must be characterized by the ability to both decompose and grow in an environment with a high concentration of oxalate, as well as by resistance to antibiotic therapy.

As with *Lactobacillus*, the ability of *Bifidobacterium* to decompose oxalate in vitro does not guarantee its degradation in vivo. Therefore, both animal and clinical trials should be conducted to verify the potential effectiveness of selected *Bifidobacterium* strains in reducing the risk of the development of urolithiasis. An animal study confirmed that *B. animalis* subsp. *Lactis* DSM 10140 is able to degrade oxalate. Mice with a deficiency in the hepatic alanine-glyoxylate aminotransferase (*Agxt^−/−^*, mouse model for primary hyperoxaluria) and wild-type mice after probiotic treatment, who were fed an oxalate-supplemented diet, showed increased ability to degrade dietary oxalate, thus limiting its absorption across the intestine and reducing urinary oxalate excretion. *B. animalis* subsp. *Lactis* DSM 10140 colonization was at a higher level in wild-type mice than in knockout mice. However, there were no significant changes to net oxalate secretion from the intestine in colonized animals. Therefore, these results suggest that colonization with *B. animalis* subsp. *Lactis* decreases urinary oxalate excretion by degrading dietary oxalate, thus limiting its absorption across the intestine, but that it does not promote enteric oxalate excretion, as in the case of *O. formigenes*. [104]. Hatch et al. [105] showed that the combined administration of the human *Oxalobacter* strain, HC-1, and *B. animalis* reduced urinary oxalate excretion in *Agxt* knockout mice compared with the same mice before therapy. However, mice that received the combined inoculum were characterized by 16% less urinary oxalate excretion than those that received HC-1 alone, which confirms the results indicating that the *Oxalobacter* strain is the best for decomposing oxalate. Additionally, the obtained results indicated that the inclusion of these strains in probiotic mixture may adversely affect their ability to break down oxalate. Therefore, paradoxically, a composition consisting of different strains, each of which individually exhibits oxalate metabolizing activity, may prove ineffective [105].

Unfortunately, only one clinical trial has been conducted to date. The obtained results showed that treatment with a mix of probiotics (*L. acidophilus*, *L. plantarum*, *L. brevis*, *S. thermophilus*, and *B. infantis*) caused a significant reduction in the excretion of oxalate in all six patients with idiopathic calcium oxalate urolithiasis and mild hyperoxaluria. Therefore, the proposed mixture of probiotics may prove effective for the prevention of urolithiasis. However, in order to unequivocally evaluate the effectiveness of the tested mixture, it is necessary to conduct these studies on a much larger population. [80]. 

A summary of previous studies using *Bifdobacterum* spp. for the prevention of urolithiasis is presented in Table 7.

## 5. Other Bacteria Associated with Oxalate Metabolism

*Eubacterium* is the second most common bacterial genus found in the gastrointestinal tract in humans, after *Bacteroides*. *E. genus* is anaerobic and produces nonspore-forming, Gram-positive rods. Ito and colleagues [106] isolated *E. lentum* WHY-1 from the feces of a Japanese male and found that it could be used to reduce the oxalate content of tea in vitro [106]. Moreover, further in vitro studies showed that *E. lentum* WYH-1 decomposed 100% of 1 mg/mL oxalate in an artificial intestinal juice [107]. Intestinal juice contains bile salts which have an antibacterial effect. Bile salt compounds destroy bacterial membranes, denature proteins, chelate iron and calcium, cause oxidative DNA damage, and control the expression of eukaryotic genes involved in host defense and immunity. Consequently, the ability of *E. lentum* to multiply and degrade oxalate in the presence of bile salts indicates the clinical applicability of these bacteria [106,107]. Unfortunately, no studies to date have confirmed the ability of *E. lentum* to degrade oxalate in vivo.

*Enterococcus faecalis* is a Gram-positive facultative anaerobe, occurring as single cocci or in chains of various lengths. *E. faecalis* is an intestinal commensal; however, it is also a significant opportunistic pathogen, causing urinary tract and wound infections [108]. Moreover, *E. faecalis* is called “generalist oxalotroph”, since it only utilizes oxalate as a carbon source in the absence of alternative energy supplies [109]. Hokama et al. [109] found that *E. faecalis*, isolated from human stool under anaerobic conditions, showed oxalate-degrading ability in vitro. Moreover, a protein analysis confirmed that oxalate-degrading *E. faecalis* produced three proteins with molecular weights of 65, 48, and 40 kDa that were not produced by nonoxalate-degrading *E. faecalis*. Two of these proteins were homologues of oxalyl-coenzyme A-decarboxylase (65 kDa) and formyl-coenzyme A-transferase (48 kDa), which are found in *O. formigenes* [109]. Interestingly, these proteins were not expressed under conditions where the oxalate-degrading strains of *E. faecalis* had access to other energy sources. Therefore, the active expression of the detected proteins is a mechanism induced by unfavorable environmental conditions which enable the survival of bacteria that only have access to oxalate as an energy source [109]. Due to the fact that *E. faecalis* is an opportunistic pathogen that can use oxalate as an alternative energy source, the clinical application of this bacterium as a probiotic for reducing the risk of developing stones in the urinary tract is severely limited.

Similar to *E. faecalis*, homologues of the genes characteristic of *O. fomingenes* have been identified in *Escherichia coli*. *YfdW* from *E. coli* is a formyl-CoA transferase [110] and *YfdU* is an oxalyl-CoA decarboxylase [111,112]. The presence of these genes suggests the ability of *E. coli* to degrade oxalate. Unfortunately, studies conducted to date have not confirmed that *E. coli* bacteria are capable of decomposing oxalate. On the other hand, certain freshly isolated fecal *E. coli* strains initially showed improved growth on an oxalate-supplemented medium, but this phenotype was soon lost upon further subculturing, which significantly limits the clinical application of these bacteria in patients with kidney stones [113].

## 6. *B. subtilis* and *L. plantarum* as Novel Recombinant Approaches

The ability to prevent the formation of urinary stones is also demonstrated by *B. subtills*. Al and colleagues [114], using the *Drosophila melanogaster* model of urolithiasis as a high-throughput screening platform to evaluate the therapeutic potential of oxalate-degrading bacteria in calcium oxalate nephrolithiasis, found that *Bacillus subtilis 168* (BS168) is promising candidate for the prevention of oxalate-induced microbiota dysbiosis. The studied BS168 was characterized by preferential growth in high oxalate concentrations and an ability to rapidly and stably colonize the *D. melanogaster* intestinal tract for as long as five days. These results were confirmed by in vitro study, which demonstrated that established MDCK renal cells treated with BS168 showed reduced adhesion and aggregation of calcium oxalate crystals. Thus, BS168 could represent a novel therapeutic adjunct to reduce the incidence of recurrent calcium oxalate nephrolithiasis in high-risk patients [114].

Another possible solution to excessive oxalate deposition is the use of pure enzyme products derived from bacteria. Such solutions allow the use of human pathogenic bacteria for the production of desired enzymes. The first reports on the application of enzymes only, rather than whole bacteria, supported the use of *B. subtilis* oxalate decarboxylase (OxDC) enzyme. This enzyme was expressed in *E. coli* and then purified [115,116]. Next, the enzyme crystals were crosslinked with glutaraldehyde and administered at a daily dose of 200 mg for 16 days to mice suffering from hyperoxaluria (mouse model with *Agxt* gene mutation). The used preparation reduced the fecal and urinary oxalate concentrations of the mice by 72% and 44% respectively, compared with a placebo group [116]. Interestingly, the administration of ethylene glycol, which induced nephrocalcinosis and kidney failure, and 80 mg/day OxDC- CLEC (cross-linked formulation of oxalate-decarboxylase) caused a 40% reduction of urinary oxalate and prevented the development of ethylene glycol-induced kidney pathology [116]. Cowley et al. [117] proposed to modify the *B. subtilis* enzyme by replacing the cysteine residue at position 383 with serine. This modification was intended to prevent protein aggregation while maintaining the proper functionality of the enzyme [117]. Importantly, OxDC exhibits optimal activity at acidic pH (3.5–5.0), and requires the presence of catalytic amounts of molecular oxygen. Due to its low pH activity, OxDC can degrade oxalate in the stomach, thus reducing the level excreted in the urine; as such, it can effectively reduce the risk of developing urolithiasis [117]. Moreover, toxicity analyses confirmed the safety of the modified formulation, and preliminary clinical studies confirmed the potential of the modified enzyme to reduce urinary oxalate [118]. Another solution to the problem of oxalate degradation is the use of recombinant probiotic bacteria. For this purpose, the probiotic bacteria that have the best ability to colonize the intestines should be selected, and the gene encoding the oxalate-degrading enzyme should be introduced into their genetic material [119]. A previous study suggested that *L. plantarum* NC8 could be used as a bacterium into which the vector with the *OxDC* gene will be introduced. *L. plantarum* NC8 is a probiotic bacterium recognized by the FDA as GRAS that does not normally express oxalate decarboxylase [120]. However, the creation of good recombinants is difficult. Previous studies confirmed that in the case of recombinants, it is important to select the appropriate promoter that determines the expression of the desired gene. Studies on the appropriate *OxDC* introducing vector have shown that the recombinants obtained may or may not secrete the enzyme on the cell outside. The first data indicated *OxDC* gene cloning into a shuttle vector in which expression was regulated by a sakacin-P-inducible promoter (pSIP high-level expression system), and that the production of an active recombinant OxDC protein is intracellular [121]. On the other hand, Sasikumar and colleagues [122] proposed the introduction into the p-SIP vector of both OxDC and homologous peptide sequences (Lp_0373 or Lp_3050), which allowed extracellular secretion of the functional enzyme by the obtained recombinant. The recombinants gained in this approach were able to degrade 50% of the oxalate present in the medium [122,123,124]. In the next study, the pSIP409 vector was used, in which the inducible promoter (P orfX) was replaced with a constitutive promoter (P ldhL), and the gene *gusA* was replaced with *oxdC*. As a consequence, recombinant *L. plantarum* which was capable of secreting dehydrogenase and degrading 90% of the oxalate contained in the medium, compared to 15% by the wild type, was obtained [125]. Moreover, animal studies confirmed that rats treated with recombinant strains of *L. plantarum*, i.e., WCFS1OxdC (strain secretes the functional OxdC outside cells) and NC8OxdC (strain nonsecretes the functional OxdC outside cells), were characterized by reduced urinary oxalate excretion and crystal deposition of calcium oxalate due to increased intestinal oxalate degradation [122,126].

## 7. Conclusions

The increase in the incidence of nephrolithiasis and the high rate of relapse have emphasized the need to develop effective methods to prevent the onset of this condition. Currently, patients with oxalate stone formation are only advised to follow an appropriate diet. Therefore, in recent years, there has been increasing interest in probiotic bacteria which are capable of degrading oxalate. A comparison of the most studied probiotics in terms of the prevention of urolithiasis is presented in the Table 8. Most research to date has focused primarily on *O. fomingenes*, which is an absolute oxalotroph with high sensitivity to antibiotics and the conditions of probiotic formulation processes, such as low pH and oxygen conditions. Therefore, the potential to use *O. formingens* for the prevention of urolithiasis is limited. Nonetheless, the undoubted advantage is that of all studied bacteria, *O. formigenes* has the highest oxalate-degrading capacity. Interestingly, later studies indicated the existence of *O. formigenes* strains characterized by resistance to low pH and the presence of oxygen, which confirmed the potential for the clinical use of *O. formigenes*. Therefore, it is necessary to conduct extensive research to select strains which are characterized by low levels of sensitivity to the aforementioned conditions. Unfortunately, as stated above, the use of *O. formigenes* is not effective in patients undergoing recent or current antibiotic therapy. A possible solution to the problem of antibiotic sensitivity could be the use of a mixture of probiotics, which would partially eliminate the problem of intestinal colonization. Reports have suggested that the use of a mixture of various probiotics may also be a good solution owing to the participation of the complete intestinal microflora in the degradation of oxalate and the reduction of its excretion in urine. Moreover, the use of a mixture of probiotic bacteria containing *Lactobacillus* or *Bifidobacterium* strains increases the degree of similarity of effective intestinal colonization. In patients with urolithiasis, the high concentration of oxalate in the intestine significantly restricts the growth and multiplication of most known probiotic bacteria, as only a few strains can obtain energy from oxalate. Therefore, the use of a mixture of bacteria with different growth potentials under conditions of high oxalate concentrations will result in the growth of bacteria which are capable of metabolizing oxalate in the initial stage, and when its concentration decreases due to the activity of those bacteria, the remaining probiotic bacteria will start to develop. Therefore, supplementation with a mixture of microorganisms can effectively restore the microbiological balance and provide effective prevention against urolithiasis. Unfortunately, to our knowledge, no research has interpreted disturbances of the intestinal microflora as a whole. Moreover, it should be emphasized that most human studies have numerous limitations. The vast majority of such studies are based on small research groups, i.e., a dozen or so people, who are often on an uncontrolled diet. Studies on *Lactobacillus* spp. and *Bifidobacterium* spp. are largely limited to in vitro and animal studies, which already indicate differences in the ability of these bacteria to metabolize oxalate in vitro and in vivo. Therefore, there is a need to conduct further studies under strictly controlled conditions involving a larger population.

## Figures and Tables

**Table 1:** Risk factors of nephrolithiasis development.

Type of Risk Factors	Risk Factors	Description	References
Intrinsic factors	Gender	Deposits in the urinary tract were found to be 2–3 times more common in men than in women.	[6]
	Age	The incidence of nephrolithiasis has increased dramatically over the past 30 years.	[1]
	Ethnic differences	Non-Hispanic white individuals were characterized by the highest stone risk (10.3%), followed by Hispanics (6.4%) and non-Hispanic African Americans (4.3%).	[17]
	Family or personal history	If someone in your family has had kidney stones, you are more likely to develop them than someone without a family history.	[18]
Extrinsic factors	Environmental factors	Regions with higher average temperatures in the United States showed the highest risk of urinary tract stone occurrence.	[19,20]
	“Western diets”	“Western diets” are characterized by the consumption of large amounts of animal protein, which leads to an increase in the excretion of calcium, oxalate, and uric acid in the urine, consequently predisposing individuals to kidney stones.	[1,2]
	Fluid intake	The reduction of fluid intake may contribute to increased urine saturation.	[21]
	Sodium intake	Increased sodium intake causes increased urine calcium and reduced citrate excretion.	[21]
	Calcium intake	Reduction in calcium intake causes an increase in urinary oxalate excretion.	[21]
	Meat intake	Increased meat intake causes a decrease in urine pH and an increase urinary calcium excretion.	[21]
	Fruits intake	Decreased fruit intake causes a decrease in urine pH and reduces in citrate excretion.	[21]
	Diet content in oxalate foods	Increased oxalate in food contributes to an increase in urinary oxalate extraction.	[21]
	BMI (body mass index)	An increase in BMI was positively correlated with increased risk of nephrolithiasis.	[1]
	Metabolic disorders	Diabetes mellitus, obesity and metabolic syndrome may increase the risk of kidney stones.	[21]
	Urinary tract infections	Patients who suffer from chronic urinary tract infections may form larger stones.	[21]

**Table 2:** Characteristics of *Oxalobacter formigenes*.

*Oxalobacter formigenes*	
Characteristic	Description
Morphology	Rod shaped
Gram staining	Gram-negative
Mobility	Nonmotile
Spore-forming	Nonspore-forming
Anaerobic/aerobic	Obligate anaerobes

**Table 3:** A summary of previous studies on the use of *O. formigenes* for the prevention of urolithiasis.

Author	Type of Study	Study Design	Main Finding
Sidhu et al. [53]	Animal study	Laboratory rats known to be noncolonized were colonized with live bacteria or treated with a preparation of oxalate-degrading enzymes derived from *O. formigenes* to test for any subsequent increase in resistance to high oxalate challenge.	The absence of *O. formigenes* in the gut increases the risk for hyperoxaluria and recurrent kidney stone disease. Replacement therapy is an efficient procedure to prevent hyperoxaluria and its complications.
Sidhu et al. [54]	Animal model of severe hyperoxaluria	Male Sprague-Dawley rats were divided into six subgroups: group 1 was given a normal diet, group 2 was given an oxalate-rich diet; group 3 was given an oxalate-rich diet and an esophageal gavage of 1 × 10^3^ *O. formigenes* each feeding for a two-week period; group 4 was given an oxalate-rich diet and an esophageal gavage of 1 × 10^5^ *O. formigenes* each feeding for a two week period; group 5 was given an oxalate-rich diet and an esophageal gavage of 1 × 10^7^ *O. formigenes* each feeding for a two week period; and group 6 was given an oxalate-rich diet and an esophageal gavage of 1 × 10^9^ *O. formigenes* each feeding for a two week period. Urinary samples were also collected to assess the level of oxalate extraction.	Urine oxalate levels were lower in the group that received an oxalate-rich diet and *O. formigenes* compared to the group that received only an oxalate-rich diet. The amount of the decrease proved to be directly proportional to the dose of bacteria.
Hatch et al. [48]	Animal study	Male Sprague–Dawley rats were divided into two groups: group 1 was colonized by esophageal gavage of a 1.5 mL inoculum of 20 × 10^8^ bacteria from a 24-h culture of a wild rat strain of *O. formigenes*, while group 2 was noncolonized. Both groups of rats were fed the same diet. Urinary samples were also collected to assess the level of oxalate extraction.	Rats colonized by *O. formigenes* were characterized by decreased urinary oxalate levels as compared with noncolonized rats on the same diet
Duncan et al. [51]	Clinical study (Four healthy volunteers)	Adult volunteers lacking detectable oxalate-degrading activity in feces were subjected to an oxalate loading test and then administered 500 mg wet weight containing approximately 10^8^ viable cells in 1 mg of *O. formigenes* strain HC−1. Finally, stool samples were collected to assess the presence of *O. formigenes*. Determination of *O. formigenes* was carried out by culture as well as PCR. Urinary samples were also collected to assess the level of oxalate extraction.	*O. formigenes* intake may reduce urinary oxalate levels in patients on a diet high in calcium oxalate.
Hoppe et al. [52]	Clinical study (16 patients with urolithiasis)	Patients were divided into two groups. The first group included nine patients to whom *O. formigenes* was administered as a frozen cell paste containing 1 g live cells equivalent to >10^10^ CFU. The second group included seven patients to whom *O. formigenes* was administered as two enteric-coated capsules per dosing (137 mg of lyophilized bulk powder of freeze-dried live cells equivalent to ~10^7^ CFU). Urine and plasma samples were collected to assess the level of oxalate extraction.	*O. formigenes* intake can reduce the urinary oxalate levels in patients with urolithiasis.
Siener et al. [55]	Clinical study (37 patients with idiopathic calcium oxalate stone)	The presence of *O. formigenes* in the feces was determined to classify patients into colonized and noncolonized groups. Determination of *O. formigenes* was carried out by culture as well as PCR. Venous blood samples were obtained in the morning after an overnight fasting period, while 24-h urine samples were collected to assess baseline oxalate urinary excretions. The patients’ diets were not controlled.	Plasma oxalate concentrations were significantly higher in noncolonized (5.79 μmol/L) than colonized stone formers (1.70 μmol/L). Colonization with *O. formigenes* was significantly inversely associated with the number of stone episodes. *O. formigenes* colonization was shown to decrease urinary oxalate excretion.
Kaufman et al. [56]	Clinical study (247 patients with recurrent calcium oxalate stones and 259 controls)	Stool samples were collected to assess the presence of *O. formigenes*. Determination of *O. formigenes* occurrence was carried out by culture as well as PCR. Urinary samples were also collected to assess the level of oxalate extraction. During the study, patients were on whatever diet they reported in the survey.	The prevalence of *O. formigenes* was 17% among case-patients and 38% among control subjects. Moreover, the colonization with *O. formigenes* was associated with a 70% reduction in the risk of recurrent calcium oxalate stone formation.
Troxel et al. [49]	Clinical study (five first time calcium stone formers and10 control participants)	Stool samples were collected for culture and detection of *O. formigenes* by PCR. Urinary samples were also collected to assess the level of oxalate extraction. Additionally, all participants underwent standard metabolic testing to evaluate their risk for recurrent stone formation.	Urine oxalate levels were lower in *O. formigenes*-positive patients compared with *O. formigenes*-negative patients (29.4 mg/day vs. 41.7 mg/day).
Kwak et al. [57]	Clinical study (30 healthy volunteers and 38 patients with urolithiasis)	Determination of *O. formigenes* presence in stool samples was carried out by PCR. Patients’ diets were not controlled.	The colonization rate of *O. formigenes* in patients with urolithiasis was significantly lower than in healthy volunteers known to be free from urolithiasis.
Kodama et al. [58]	Clinical study (55 male and 37 female healthy volunteers)	Determination of the presence of *O. formigenes* in stool samples was carried out by PCR and culture-based method. Participants’ diets were not controlled.	Female subjects showed a 15% lower rate of *O. formigenes* occurrence than males.
Kumar et al. [59]	Clinical study (63 patients with calcium oxalate stone formers and 40 controls from North India)	Stool samples were collected to assess the presence of *O. formigenes*. Determination of *O. formigenes* was carried out by PCR and Southern blotting. Urinary samples were also collected to assess the level of oxalate extraction.	*O. formigenes* was present in 65% of normal individuals and 30% of calcium oxalate stone formers. Colonies were present in only 5.6% of patients with three or more stone episodes. Oxalate excretion was lower in patients colonized with *O. formigenes* compared to those with no colonization.
Kharlamb et al. [60]	Clinical study (patients with confirmed *O. formigenes* colonization)	The impact of antibiotics (amoxicillin and clarithromycin) on *O. formigenes* colonization was compared in two groups: group 1 received antibiotics for gastric infection with *Helicobacter pylori*, while in group 2 without *H. pylori*, subjects were not receiving antibiotics. *O. formigenes* colonization in stool was detected by oxalate degradation at baseline and after one and six months.	Among the 12 patients who were positive for *O. formigenes* who did not receive antibiotics, 11 (92%) had *O. formigenes*, as evaluated through stool tests at one and six months. Of the 19 participants who were positive for *O. formigenes* and who received antibiotics for *H. pylori*, only seven (36.8%) continued to be colonized by *O. formigenes* on follow-up stool testing at one and six months.
Nazzal et al. [62]	Clinical study (23 patients with a positive test of *H. pylori* and 46 controls)	Patients with confirmed *H. pylori* infection received antibiotics (amoxicillin and clarithromycin), while control groups did not. Fecal samples were examined for the presence of *O. formigenes* and for microbiota characteristics. Determination of *O. formigenes* was carried out by PCR. Urine, collected after serially fasting and following a standard meal, was tested for oxalate and electrolyte concentrations.	*O. formigenes* colonization was significantly suppressed in antibiotic-exposed subjects but remained stable in controls.
Jiang et al. [63]	Clinical study (11 *O. formigenes* positive and 11 *O.formigenes* negative nonstone forming adults)	The study was divided into two, three-week dietary phases. For the first phase, dietary oxalate intake was varied, including 50 mg daily for the first week, 250 mg for the second week, and 750 mg for the third week. For the second phase, dietary calcium intake was varied, i.e., 400 mg daily for the first week, 1000 mg for the second week, and 2000 mg for the third week. Finally, urine and stool samples were collected and used to determine stone risk parameters and *O. formigenes* levels.	*O. formigenes* levels increased 10-fold as dietary oxalate increased 15-fold, while there was a decrease in *O. formigenes* content with increasing calcium intake.
Hoppe et al. [69]	Phase I/II clinical trial (28 patients randomized to the treatment group (OC5) or the placebo group	There was no significant difference in the change in urinary oxalate excretion and plasma oxalate excretion between the studied groups after eight weeks of OC5 treatment. However, the group which received OC5 treatment was characterized by increased urinary oxalate excretion compared to urinary creatinine excretion.	Treatment with OC5 preparation did not significantly reduce urinary or plasma oxalate extraction; however, this therapy was well tolerated and successfully delivered to the gastrointestinal tract.
Milliner et al. [68]	Phase II/III clinical trial (26 patients randomized to the treatment group (OC3) or the placebo group	There were no significant differences in the change in urinary oxalate excretion, urinary oxalate/urinary creatinine ratio or plasma oxalate excretion among the studied groups after 24 weeks of OC3 treatment; however, this treatment was well tolerated.	OC3 treatment was well tolerated but was not found to reduce urinary oxalate excretion.

**Table 4:** Characteristics of *Lactobacillus* spp.

*Lactobacillus* spp.	
Characteristic	Description
Morphology	Rod shaped
Gram staining	Gram-positive
Mobility	Nonmotile
Spore-forming	Nonspore-forming
Anaerobic/aerobic	Facultative anaerobic
Catalase	Catalase-negative

**Table 5:** A summary of previous studies on the use of *Lactobacillus* spp. for the prevention of urolithiasis.

Author	Type of Study	Study Design	Main Finding
Turroni et al. [81]	In vitro	Among the 60 *Lactobacillus* strains analyzed, 32 belonged to the *L. acidophilus* group, 6 to *L. gasseri*, 7 to *L. plantarum*, 3 to *L. casei*, 2 to *L. rhamnosus*, 1 to *L. salivarius*, 1 to *L. johnsonii*, 2 to *L. paracasei*, 1 to *L. delbrueckii* subsp. *Lactis*, 1 to *L. delbrueckii* subsp. *Bulgaricus*, 2 to *L. brevis*, 1 to *L. reuteri*, and 1 to *L. helveticus*. All strains were grown in an oxalate-supplemented media to evaluate the ability of the bacteria to degrade oxalate. The identification of *oxc* and *fcr* was performed with PCR.	High oxalate degradation levels were obtained with *L. casei* LC11 (48%) and *L. rhamnosus* PB41 (47%). All the other tested strains exhibited a markedly lower oxalate-degrading activity, ranging from 0% to 20%. The homologues of the *oxc* and *fcr* were identified in *L. acidophilus* LA14.
Turroni et al. [82]	In vitro	The oxalate-degrading activities of 14 *Bifidobacterium* strains (*B. adolescentis* ATCC 15703, *B. animalis* subsp. *Lactis* DSM 10140, BA30, Bb12, BI07, and L15, *B. bifidum* S16, *B. breve* ATCC 15700 and BBSF, *B. catenulatum* B665, *B. longum* biotype *longum* S123, ATCC 15707, and W11, and *B. longum* biotype *suis* ATCC 27533), cultured in oxalate-rich media, was measured by a capillary electrophoresis technique. The identification of the *oxc* gene was performed with PCR	Only the five *B. animalis* subsp. *Lactis* strains (DSM 10140, BA30, Bb12, BI07, and L15) showed oxalate-degrading activity with 100% of oxalate consumption after 5 days of incubation, whereas no degrading activity was exhibited by all the other bifidobacterial strains tested.
Mogna et al. [83]	In vitro	Thirteen strains of *Lactobacillus*(*L. paracasei*, *L. gasseri* LGS01, LGS02, *L. acidophilus* LA07, LA02, *L. plantarum* LP01, *L. reuteri* LRE03, LRE02, *L. rhamnosus* GG, LR06, *L. reuteri* LRE04, LR06, *L. delbrueckii* subsp. *Delbrueckii* LDD01) and five *Bifidobacterium* (*B. breve* BR03, *B. animalis* DSM 20104, *B. longum* BL02, BL03, *B. lactis* BA05) were cultured in an ammonium oxalate-supplemented medium and their oxalate-degrading activity was tested by a novel HPLC method.	Screening of different *Bifidobacterium* strains for their in vitro ability to metabolize oxalates showed that *B. breve* BR03 degraded 28.2% of oxalate, *B. animalis*—27.7%, *B. longum* BL03—25.3%, *B. lactis* BA05—15.5%.
Lewanika et al. [71]	In vitro	*L. gasseri* AM63 was grown anaerobically in a medium supplemented with sodium oxalate. The oxalate-degrading capacity of *L. gasseri* was measured by commercial oxalate enzymatic kit assay. The identification of *oxc* and *frc* orthologs was performed by PCR.	*L. gasseri* AM63T ability to degrade oxalate was confirmed. Molecular analysis confirmed the presence of orthologs of the *oxc* and *fcr* genes in the genome of *L. gasseri* AM63T.
Azcarate-Peril et al. [84]	In vitro	Strains of *L. acidophilus* were grown in a medium supplemented with sodium oxalate. The identification of *oxc* and *frc* was performed by PCR.	*Oxc* and *frc* expression were induced as an operon in the presence of oxalate under acid conditions.
Gomathi et al. [86]	In vitro	Six hundred and seventy-three bacterial isolates were isolated from stool samples collected from 30 patients. A strain analysis showed that 251 of the isolates were lactic acid bacteria, but only 17 were capable of metabolizing oxalate. The bacteria selected in this way were analyzed to assess their ability to adhere to epithelial cells.	Obtained results suggest that *L. fermentum* TY5, *L. fermentum* AB1, and *L. salivarius* AB11 may degrade an oxalate and are characterized by significant adhesion to epithelial cells and strong antimicrobial activity. Therefore, these strains may serve as good probiotic candidates for preventing hyperoxaluria.
Giardina et al. [87]	In vitro	Eleven strains of lactic acid bacteria (*Lactobacillus* and *Bifidobacterium*), already included in the list of bacteria which are safe for human use, were investigated for their ability to degrade oxalate, by means of the RP-HPLC-UV method, and modulate inflammation in an in vitro model system based on peripheral blood mononuclear cells.	*L. plantarum* PBS067, *L. acidophilus* LA-14, *B. breve* PBS077, and *B. longum* PBS078 were able to degrade an oxalate in conditions of hyperoxaluria and the inflammatory events associated with the oxalate accumulation.
Campieri et al. [80]	In vitro and animal	*L. acidophilus*, *L. plantarum*, *L. brevis*, *S. thermophilus*, *B. infantis* were grown under anaerobic conditions in an oxalate-supplemented media to evaluate their ability to degrade oxalate. Next, the mixture of freeze-dried bacteria was administered to six patients with idiopathic calcium oxalate urolithiasis and mild hyperoxaluria for a period of four weeks. Finally, urinary samples were collected to assess the level of oxalate extraction.	All the tested bacteria showed an oxalate degradation capacity of 1–11%. The mixed probiotic treatment resulted in a great reduction of the excretion of oxalate in all six patients.
Wei et al. [90]	In vivo (ethylene glycol induced-animal model of kidney stones)	Male rats were given 1% ethylene glycol dissolved in their drinking water for four weeks to develop hyperoxaluria, and half of them received an additional LPN1 for four weeks prior to treatment with ethylene glycol as a preventive intervention.	LPN1 probiotic can prevent ethylene glycol induced hyperoxaluria by regulating the gut microflora and improving gut barrier function.
Lieske et al. [92]	Clinical (10 patients with chronic fat malabsorption, calcium oxalate stones, and hyperoxaluria)	Patients took 1 (4 g), 2 (8 g), and 3 (12 g) packets of Oxadrop^®^ daily for three four-week periods. The preparation was mixed in a glass of cold beverage including water, orange juice, or tea, but no milk. The preparation was taken 1 to 2 h after the major meal of the day. Finally, urine samples were collected and it has been determined urinary concentrations of oxalate.	70% of patients were characterized by decreased urinary oxalate extraction. Moreover, taking 4 g of Oxadrop^®^ per day reduced urinary oxalate excretion by 19%, and this increased to 24% when 8 g per day were administered.
Lieske et al. [93]	Clinical (40 patients with nephrolithiasis and mild hyperoxaluria of unknown etiologist)	Patients were divided into three study groups that received placebo, Agri-King Synbiotic Preparation, and Oxadrop^®^, respectively. Finally, urinary samples were collected to assess the level of oxalate extraction.	Tested probiotic preparation did not influence urinary oxalate levels in patients on a restricted oxalate diet.
Goldfarb et al. [94]	Clinical (20 stone formers with idiopathic and enteric hyperoxaluria)	Patients were divided into two studied groups that received placebo and Oxadrop^®^, respectively. Finally, urinary samples were also collected to assess the level of oxalate extraction.	Oxadrop^®^ did not reduce urinary oxalate excretion in participants with idiopathic hyperoxaluria as compared with the placebo group.
Siener et al. [95]	Clinical (20 healthy subjects)	Healthy volunteers who were initially on a normal diet changed to a supplemented diet with calcium oxalate for six weeks and received lactic acid bacteria preparation for five weeks. After this time, they returned to their original diet. Urine samples were collected weekly throughout the study period. Moreover, blood samples were analyzed before and at the end of treatment.	The study preparation neither reduced urinary oxalate excretion nor plasma oxalate concentration.
Ferraz et al. [96]	Clinical (14 stone-forming patients without hyperoxaluria)	Patients consumed a diet supplemented with oxalate for four weeks and a lyophilized *L. casei* and *B. breve* preparation was given after meals during the last two weeks. Finally, urinary samples were collected to assess the level of oxalate extraction.	Seven out of 14 patients presented a reduction in oxaluria after probiotic preparation as compared before treatment, being the reduction higher than 25% in four participants and up to 50% in two participants.
Okombo et al. [88]	Clinical (11 healthy volunteers)	Participants took VSL#3^®^ for four weeks followed by a four-week washout period. Oxalate load tests, providing a total of 80 mg oxalate, were conducted at baseline (pre-probiotic), and after the probiotic and washout periods. Fecal samples were collected before the initiation of the study to assess the presence of *O. formigenes*. Finally, urinary samples were collected to assess the level of oxalate extraction.	The average total 22 h oxalate absorption at baseline (30.8%) was higher compared to after the probiotic (11.6%) and washout (11.5%) periods.
Al-Wahsh et al. [97]	Clinical (11 healthy nonstone formers)	Healthy nonstone formers divided into three groups: (i) oral ingestion of sodium oxalate (176 mg), (ii) 2 packets of VSL#3^®^ preparation with a 176 mg oxalate sodium, (iii) 1 packet of VSL#3^®^ preparation with a 176 mg oxalate sodium. Finally, urinary samples were collected to assess the level of oxalate extraction.	Both the doses of VSL#3^®^ were effective in reducing urinary oxalate and estimated oxalate absorption with no significant difference between the two probiotic doses.
Kwak et al. [89]	Stone-forming animal model using selective cyclo-oxygenase 2 inhibitor	Male Sprague–Dawley rats divided into seven groups: (i) rats were maintained on the powdered regular diet for the whole study, (ii) rats received the powdered regular diet supplemented with 3% (*w*/*v*) sodium oxalate, (iii) rats were maintained on the powdered regular diet supplemented with 3% sodium oxalate for the whole study with each rat receiving 1 mL of celecoxib (100 mg/kg) for the first eight days, (iv) rats were maintained on the powdered regular diet supplemented with 3% sodium oxalate for the whole study with each rat receiving 1 mL of celecoxib (100 mg/kg) for the first eight days and 10% (*w*/*v*) skim milk, (v) rats were maintained on the powdered regular diet supplemented with 3% sodium oxalate for the whole study with each rat receiving 1 mL of celecoxib (100 mg/kg) for the first eight days and *L. casei* HY2743 (5 × 10^8^ CFU/mL diet), (vi) rats were maintained on the powdered regular diet supplemented with 3% sodium oxalate for the whole study with each rat receiving 1 mL of celecoxib (100 mg/kg) for the first eight days and *L. casei* HY7201 (5 × 10^8^ CFU/mL diet), (vii) rats were maintained on the powdered regular diet supplemented with 3% sodium oxalate for the whole study with each rat receiving 1 mL of celecoxib (100 mg/kg) for the first eight days and *Lactobacillus casei* HY2743, *L. casei* HY7201 and 10% (*w*/*v*) skim milk. Finally, urinary samples were also collected to assess the level of oxalate extraction and all rats were sacrificed and morphologic examination involving crystal formation was observed under a microscope.	In both groups of co-treatment and previous treatment with *L. casei* HY2743 and *L. casei* HY7201, urine oxalate excretion decreased compared to the group without *Lactobacillus*. The dissecting microscope examination of kidneys in the rats in two previous treatment groups and the co-treatment group with *L. casei* HY7201 showed less abundant crystals than control groups.

**Table 6:** Characteristics of *Bifidobacterium* spp.

*Bifidobacterium* spp.	
Characteristic	Description
Morphology	Bifid or irregular V- or Y-shaped rods resembling branches
Gram staining	Gram-positive
Mobility	Nonmotile
Spore-forming	Nonspore-forming
Anaerobic/aerobic	Anaerobic
Catalase	Catalase-negative

**Table 7:** A summary of previous studies on the use of *Bifdobacterum* spp. for the prevention of urolithiasis.

Author	Type of Study	Study Design	Main Finding
Federici et al. [103]	In vitro	Twelve strains of *Bifidobavterium* were grown under anaerobic conditions in an oxalate-supplemented media to evaluate the ability of the bacteria to degrade oxalate. The identification of *oxc* was also performed through PCR and Western Blotting. The activity of the oxalyl-CoA decarboxylase was measured capillary electrophoresis.	*Oxc* was identified in the *Bifidobacterium lactis* DSM 1014, *B. dentium*, *B. gallicum*, *B. pseudocatenulatum*, and *B. pseudolongum*. Among the 12 studied bacterial strains, *Bifidobacterium lactis* DSM 10140 showed the highest oxalate-degrading activity in a preliminary screening. Moreover, the oxalate-degrading ability of certain *Bifidobacterium* species was also shown to be strain-specific.
Giardina et al. [87]	In vitro	Eleven strains of lactic acid bacteria (*Lactobacillus* and *Bifidobacterium*), already included in the list of bacteria which are safe for the human use, were investigated for their ability to degrade oxalate by means of the RP-HPLC-UV method and modulate inflammation in an in vitro model system based on peripheral blood mononuclear cells.	*L. plantarum* PBS067, *L. acidophilus* LA-14, *B. breve* PBS077, and *B. longum* PBS078 were able to degrade oxalate in conditions of hyperoxaluria and reduce the incidence of inflammatory events associated with oxalate accumulation.
Campieri et al. [80]	In vitro and animal	*L. acidophilus*, *L. plantarum*, *L. brevis*, *S. thermophilus*, and *B. infantis* were grown under anaerobic conditions in an oxalate-supplemented media to evaluate the ability of the bacteria to degrade oxalate. In the next step, a mixture of freeze-dried bacteria was administered to six patients with idiopathic calcium oxalate urolithiasis and mild hyperoxaluria for a period of four weeks. Finally, urinary samples were collected to assess the level of oxalate extraction.	All of the tested bacteria showed an oxalate degradation capacity of 1–11%. The mixed probiotics treatment resulted in a significant reduction in the excretion of oxalate in all six patients.
Mogna et al. [83]	In vitro	Thirteen *Lactobacillus* strains (*L. paracasei*, *L. gasseri* LGS01, LGS02, *L. acidophilus* LA07, LA02, *L. plantarum* LP01, *L. reuteri* LRE03, LRE02, *L. rhamnosus* GG, LR06, *L. reuteri* LRE04, LR06, *L. delbrueckii* subsp. *Delbrueckii* LDD01) and five *Bifidobacteria* (*B. breve* BR03, *B. animalis* DSM 20104, *B. longum* BL02, BL03, *B. lactis* BA05) were cultured in ammonium oxalate-supplemented medium, and then their oxalate-degrading activity was tested by a novel HPLC method.	Screening of different *Bifidobacterium* strains for their in vitro ability to metabolize oxalates showed that *B. breve* BR03 degraded 28.2% of oxalate, *B. animalis* degraded 27.7%, *B. longum* BL03 degraded 25.3%, and *B. lactis* BA05 degraded 15.5%.
Klimesova et l. [104]	Mouse model of primary hyperoxaluria	*B. animalis* subsp. *Lactis* DSM 10140 and *B. adolescentis* ATCC 15703 were administered to wild-type mice and to mice deficient in the hepatic enzyme, alanine-glyoxylate aminotransferase, that were fed an oxalate-supplemented diet. Finally, urine samples were collected to assess the level of oxalate extraction, and fecal samples were collected and the colonization status of mice with *Bifidobacterium* was monitored by PCR on weekly basis.	The administration of *B. animalis* subsp. *Lactis* led to a significant decrease in urinary oxalate excretion in wild-type and *Agxt*^−/−^ mice compared to treatment with *B. adolescentis*. The wild-type mice were characterized by a higher level of *B. animalis* subsp. *Lactis* colonization than *Agxt*^−/−^ mice.
Hatch [105]	Male and female C57BL/6 mice with *Agxt* knockout	*Agxt* knockout mice were fed a diet supplemented with oxalate during the study period. In the course of the study, a gavage inoculum containing both HC-1 and *B. animalis* was given to one of the groups and HC-1 alone was given to the other. Twelve days following the gavage procedure, when mice were confirmed as colonized, urine was collected to assess the level of oxalate extraction.	The combined administration of the human *Oxalobacter* strain, HC-1, and *B. animalis* reduced urinary oxalate excretion in knockout mice compared with the same mice before therapy. The mice that received the combined inoculum were characterized by 16% less urinary oxalate excretion than those that received HC-1 alone.

**Table 8:** Characteristics of *O. formigenes*, *Lactobacillus* and *Bifidobacterium* in the prevention of urolithiasis.

Characteristic	*O. formigenes*	*Lactobacillus*	*Bifidobacterium*
Oxalate degradability	The presence of two enzymes (formyl-CoA-transferase and oxalyl-CoA-decarboxylase) ensures the ability of bacteria to break down oxalate [46].	*Lactobacillus* strains have *oxc*-like genes which promote oxalate degradability; thus, these strains showed highly variable oxalate degrading capacity. The highest ability to degrade oxalates was demonstrated in *L. acidophilus* NCFM and *L. gasseri* AM63T; however, this capacity was lower than that of *O. formigenes* [71,81,82,83,84].	*Bifidobacterium* species, including *B. animalis* subsp. *Lactis*, *B. dentium*, *B. gallicum*, *B. pseudocatenulatum*, and *B. pseudolongum*, were characterized by the presence of *oxc* and *frc* homologues, suggesting that these bacteria may be capable of breaking down calcium oxalate. The best ability to degrade oxalates was demonstrated in *B. lactis* DSM 10140; however, this capacity was lower than that of *O. formigenes* [103].
Ability to grow in the presence of oxalate	Oxalate use as the sole source of carbon and energy causes these bacteria to show unlimited growth in conditions of high oxalate concentrations [41,42].	Under conditions of limited access to other energy sources, they are able to grow using oxalate as an energy source [70,71].	In the case of *Bifidobacterium* strains, it was shown that bacteria with a high capacity to degrade oxalate are also characterized by growth restriction at high oxalate concentrations and vice versa. The only bacteria that showed a relatively high ability to degrade oxalate and grow indefinitely at high concentrations was *B. infantis* [80].
Sensitivity to antibiotics	*O. formigenes* shows high sensitivity [44,45].	Susceptibility analyses of *Lactobacillus* bacteria showed that the bacteria were resistant to a group of 14 antibiotics, which included inhibitors of cell wall synthesis, protein synthesis, nucleic acid synthesis and cytoplasmic membrane function [127].	All strains of *Bifidobacterium* were sensitive to penicillins: penicillin G, amoxicillin piperacillin, ticarcillin, imipenem and common anti-Gram-positive antibiotics (macrolides, clindamycin, pristinamycin, vancomycin and teicoplanin) [128].
Sensitivity to low pH and the presence of oxygen	Sensitivity to low pH strongly limits the use of oral probiotics containing *O. formingens* due to transport through the acidic gastric-intestinal juice. However, the individual strains of *O. formigenes* may differ in their resistance to pH and the presence of oxygen. The persistence of unfavorable conditions favors the development of the “stationary growth advantage” (GASP) phenotype of selected strains. Moreover, due to the presence of superoxide dismutase, some *O. formigenes* strains show the ability to temporarily acclimatize to aerobic conditions [49,50].	*Lactobacillus* strains are capable of shifting from fermentative to respiratory metabolism; this was associated with an increase in biomass, long-term survival, and production of antioxidant enzymes. In turn, the sensitivity of *Lactobacillus* to low pH showed high variability: *L. gasseri* and *L. fermentum* were the most resistant to low pH, while *L. gasseri* showed the best resistance to bile acid salts [129,130].	In general, it can be considered that Bifidobacterium, with the exception of *B. animalis*, have a weak acid tolerance. However, there are some mechanisms that allow adaptation to acidic conditions, and thus ensure the survival of bacteria in gastrointestinal juice. *Bifidobacterium* shows reduced NAD-oxidase and -peroxidase activity, and reduced NAD-oxidase and -peroxidase activity was inversely correlated with their sensitivity to oxygen [131,132].
Possibility of lyophilization and production in the form of conventional probiotics	Most strains are sensitive to the conditions of lyophilization and the formulation of conventional probiotics. Moreover, the administration of *O. formigenes* in yoghurt is also very limited due to the low pH [49,50].	Most *Lactobacillus* strains are FDA GRAS, and are administered in the form of encapsulated probiotics. Therefore, these bacteria tolerate the lyophilization and formulation processes well [72].	Most *Bifidobacterium* strains are FDA GRAS and are administered in fermented dairy products. Therefore, these bacteria tolerate production processes well [131].
The optimal mode of administration	Successful and long-lasting colonization has been observed in healthy adults where *O. formigenes* was formulated as a spread on a turkey sandwich with a sodium oxalate load, while colonization of the intestines of *O. formigenes*, which was provided either in lyophilized form or as a frozen cell paste to patients with primary hyperoxaluria, was unsuccessful [51,52].	Oral preparations in the form of drops, capsules, sachets and lozenges [72].	Usually administered in fermented dairy products [131].
FDA approved as GRAS	Not certified GRAS by the FDA [46].	Certified as GRAS by the FDA [72].	Certified as GRAS by the FDA [99].
Clinical trials	Clinical trials of Oxabact^®^ OC5 (I/II phase) and Oxabact^®^ OC3 (II/III phase) did not confirm the ability of the preparations to lower urine oxalate excretion [67,68,69].	Studies on an Oxadrop^®^ probiotic preparation containing *L. acidophilus*, *B. infantis*, *S. thermophilus*, and *L. brevis* yielded conflicting results. A VSL#3^®^ supplement containing *Streptococcus thermophilus*, three *Bifidobacterium* species (*B. breve*, *B. longum*, and *B. infantis*), and four *Lactobacillus* species (*L. acidophilus*, *L. plantarum*, *L. paracasei*, and *L. delbrueckii* subsp. *Bulgaricus*) reduced urinary oxalate and increased oxalate absorption [92,93,94,95,96,97,98].	

## Data Availability

No new data were created or analyzed in this study. Data sharing is not applicable to this article.
